# Formalin Induced Micronucleus Formation in the Buccal Mucosa of Pathology Laboratory Workers

**DOI:** 10.30699/IJP.2023.1989457.3062

**Published:** 2023-10-15

**Authors:** Ghazal Akhlaghi, Fatemeh Shahsavari, Maedeh Ghorbanpour

**Affiliations:** 1 *Private Practice, Tehran, Iran*; 2 *Department of Oral and Maxillofacial Pathology, Faculty of Dentistry, Tehran Medical Sciences, Islamic Azad University, Tehran, Iran*

**Keywords:** Buccal mucosa cells, Formaldehyde, Genotoxicity, Micronucleus, Years of exposure

## Abstract

**Background & Objective:**

Formaldehyde is an irritating substance that is categorized as a definite carcinogen (Group A1), according to the International Agency for Research on Cancer (IARC). This study was conducted to determine the role of this substance in the frequency of micronuclei (MN) in the buccal mucosal cells due to long-term exposure of the pathology staff to formaldehyde.

**Methods:**

In this case-control study, 32 pathology laboratory staff members were assigned to the case group, and 32 staff members who were not exposed to formaldehyde were assigned to the control group. Buccal mucosa cells were collected with a wet spatula and stained with Papanicolaou stain. In each sample, 500 cells were counted; then, the frequency of MN and the average number of MN in the micronucleated cells were assessed and compared between the 2 groups using the independent *t* test. Furthermore, the relationship between gender and MN was evaluated using the independent *t* test. The relationship between years of exposure and time of exposure during the day (in hours) for the case group, as well as the relationship between age and frequency of MN was analyzed using the Pearson correlation coefficient.

**Results:**

The mean frequency of MN in exfoliated buccal cells was 18.33±12.36 in the case group, which was significantly higher than the control group (10.55±6.22; *P*=0.003). The difference in the mean number of total MN in the micronucleated cells was not significant between the case and control groups (*P*=0.11). The relationship between sex, age, and years of exposure with the mean frequency of MN and the total number of MN in the micronucleated cells was not significant. The relationship between exposure time during the day and both the mean frequency of MN and the total number of MN in the micronucleated cells was significant (*P*=0.03).

**Conclusion:**

Formaldehyde exposure and extended time of exposure during the day can increase the frequency of MN, which can prognosticate the incidence of precancerous and cancerous lesions. Therefore, continuous exposure to formaldehyde can be considered an occupational health hazard, though further studies are needed to confirm this result.

## Introduction

**Formaldehyde is an organic substance with a carbonyl compound, which is colorless and has a pungent, irritating gas/vapor (** 1 **). This substance has a high reactivity and high solubility in water, and that's why this compound is easily distributed in the human body. Furthermore, formaldehyde is an important precursor for many other chemical substances and compounds (** 2 **,** 3 **). In the medical field, formaldehyde is widely used in departments for sterilization in autopsy rooms and pathology/histology laboratories as a preservative (formalin) or dehydrating agent during tissue preparation and staining (** 1 **,** 2 **). Due to these properties, exposure to formaldehyde has adverse health effects, including some acute effects (such as asthma and respiratory problems) and carcinogenic effects (such as nasopharyngeal, leukemia, and sinonasal cancers) (** 2 **,** 3 **). According to the International Agency for Research on Cancer (IARC), formaldehyde is classified as a human carcinogen (Group A1). However, its effects depend on the concentration and duration of exposure (** 1 **,** 4 **-** 6 **).**

**Considering the potential of formaldehyde exposure to induce precancerous/cancerous lesions, it seems necessary to conduct screening and diagnostic studies for such lesions in those individuals who have been exposed to this substance (** 7 **). One of these screening tests is the micronucleus (MN) test in the buccal mucosa, which is very appropriate because of its advantages, such as easy and rapid cell separation (** 8 **).**

**MN is a very small nuclear body that separates from the main nucleus during the cell cycle interphase (** 9 **,** 10 **). They have complete chromosomes or parts of chromosomes (** 11 **,** 12 **). MN is one of the biological signs of genotoxicity in human red blood cells, lymphocytes, reticulocytes, and cells of the human buccal mucosa (** 13 **,** 14 **). The presence of MN is used as a criterion for measuring and diagnosing aneugenicity and clastogenicity, as well as to study the genotoxicity of various chemicals (** 9 **,** 15 **). This test is one of the most sensitive tests for DNA damage, which is used as one of the most reliable methods to investigate the pathological role of occupational and environmental factors in various human and animal epidemiological studies (** 6 **,** 13 **,** 16 **-** 18 **). The effect of formaldehyde on the formation of MN has been discussed in previous studies (** 3 **,** 19 **,** 20 **). However, many questions remain unanswered about its frequency. It is important to emphasize that although dispersed formaldehyde is rapidly metabolized, molecules that enter the cytoplasm of dividing cells can damage DNA, which in turn increases the frequency of MN (** 2 **). On the other hand, there is considerable inconsistency regarding the concentration of gas at the time of exposure and other related factors in various studies (** 21 **-** 23 **). Furthermore, there is a lack of information about the effects of formaldehyde exposure on pathology laboratory staff members in Iran; thus, we conducted this study to determine the genotoxic effects of formaldehyde regarding the duration of exposure by year and per hour in a day and other relevant factors.**

## Material and Methods

**Study Subjects**

This historical cohort study was performed in Tehran and Rasht cities, Iran, on 64 individuals who met the inclusion criteria. The individuals with a history of smoking, alcohol drinking, radiotherapy, present systemic disease, recent viral disease, consuming any drugs, and occupations related to chemical agents were excluded from the study. The eligible participants were divided into case and control groups (n=32 per group). In the case group, participants had at least 1 year of working experience and were exposed to formaldehyde at least 4 days a week in pathology laboratories (i.e., Razi Pathology Laboratory in Rasht City and Pathology Department of Faculty of Dentistry of Islamic Azad University of Tehran). In the control group, participants were unexposed employees working in administrative offices. These 2 groups were matched according to gender and age. Written informed consent was obtained from all participants. This study was approved by the Ethics Committee of Islamic Azad University of Medical Sciences (code: IR.IAU.DENTAL.REC.1399.025). 

**Sampling, Cytological Preparation, and Evaluation**

All individuals were requested to wash their mouths carefully before collecting the buccal mucosal cells. Then, the cells were scraped by a wet spatula and distributed on a small, clean glass slide for each sample. Papanicolaou staining was used to evaluate cells containing MN after the fixation procedure using the Pathofix spray (PADTAN TEB Co, Tehran, Iran) and drying at room temperature. 

**Fig 1:**
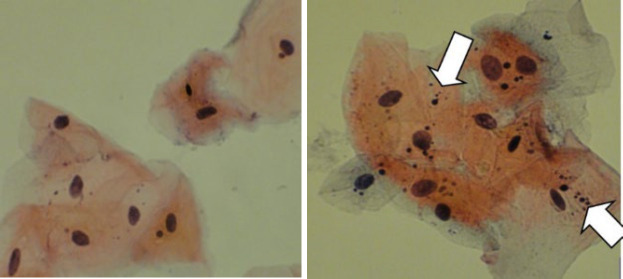
Papanicolau staining shows micronucleus (arrow) in the buccal mucosal cells of individuals working in a pathology laboratory (A) and unexposed individuals (B) (X200).

Cell examination was performed under an optical microscope (Nikon YS-100, Japan) at ×400 magnification, and the criteria applied by Tolbert *et al.* were used for the evaluation of MN (24). A total of 500 cells were counted for each sample, and presence of the cells with MN was reported in percentages. The mean number of MN in the cells containing MN was calculated and reported per cell. Figure 1 illustrates the presence of MN in the buccal cells for both the case and control groups. It should be emphasized that all microscopic glass slides were assessed by 2 calibrated oral and maxillofacial pathologists who were blinded to the specimens; in case of disagreement, the average counts were recorded for each sample.

**Statistical Evaluation**

The data were entered into SPSS version 24 (SPSS Inc., Chicago, IL., USA) after counting. Regarding the normal distribution of the data, the frequency of MN and the mean number of MN in the micronucleated cells were compared between the 2 groups using an independent *t* test. Furthermore, relationship between the gender and MN was evaluated using an independent *t *test. The relationship between years of exposure and time of exposure during the day (in hours) for the case group, as well as the relationship between age and frequency of MN, was analyzed using the Pearson correlation coefficient. P-values less than 0.05 were considered statistically significant.

## Results

**Demographic Characteristics**

The present study was conducted on 32 pathology laboratory workers (11 males and 21 females) with an average age of 35.65±3.22 years, as well as on 32 controls (11 males and 21 females) with an average age of 33.12±4.51 years. The participants in the 2 groups were matched according to age and gender.

**Mean Frequency of MN in the Exfoliated Buccal Cells of the 2 Groups**

The results showed a significant difference between the 2 groups (*P*=0.003) regarding the mean frequency of MN (Table 1).

**The Mean Number of MN in the Micronucleated Cells of the 2 Groups**

No significant differences were found in the mean number of MN in cells containing MN between the case and control groups (*P*=0.11; Table 1). 

**Table 1:** The mean frequency of micronucleus and the mean number of micronuclei in the cells containing micronucleus in buccal mucosal cells

Study Groups	Micronucleus Frequency (Percentage)				Mean Number of Micronucleus per Cell			
	Mean± SD	Min	Max	P-value	Mean± SD	Min	Max	P-value
Pathology laboratory workers	18.13±12.36	2	48.6	*P*=0.003	1.68±1.72	1.12	2.60	*P*=0.11
Non-exposed subjects	10.55±6.22	3.4	26.2		2.23±1.88	1.30	3.85	

SD: Standard Deviation, Min: Minimum, Max: Maximum

**The Relationship Between Years of Exposure and Mean Frequency of the MN in the Buccal Mucosa**

In the present study, the laboratory staff had varying levels of work experience, ranging from 1 year as the lowest to 35 years as the most extensive experience.

**Fig. 2:**
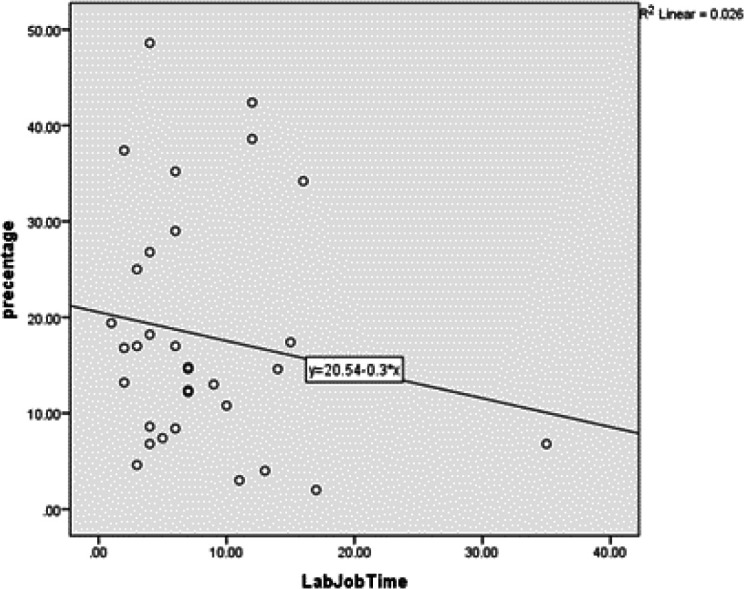
The Relationship between years of exposure and mean frequency of the micronuclei in the buccal mucosa

The Pearson correlation coefficient showed that years of exposure had no significant relationship with the average frequency of MN in the buccal mucosa (*P*=0.17). The value of the Pearson correlation coefficient was -0.07. In other words, this correlation was inverse and weak, meaning that with increasing work experience, the percentage of the cells containing MN in the buccal mucosa will decrease; however, this reduction was not significant (Table 2 and Figure 2).

**Table 2:** The relationship between years of exposure and micronuclei in the buccal mucosa

Number of MN in the Cells Containing MN		Percentage of Cells Containing MN		Years of Exposure
P-value	Mean±SD	P-value	Mean±SD	
***P*** **=0.28**	2.09±1.73	*P*=0.17	18.48±11.10	**0-10** **years**
	1.28±1.69		23.57±16.75	**10-20 years**
	0		0	**20-30 years**
	2.11±0		6.8±0	**30-40 years**

SD: Standard Deviation

**Fig. 3:**
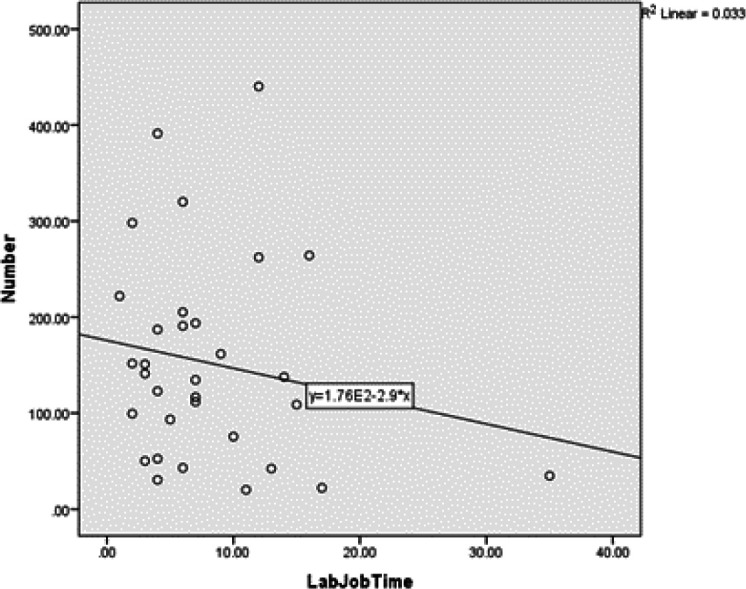
The relationship between years of exposure and mean number of micronuclei in the cells containing micronucleus

**The Relationship Between** **Years of Exposure and Mean Number of MN in the Cells Containing MN**

The Pearson correlation coefficient showed that years of exposure had no significant relationship with the number of MN in micronucleated cells (*P*=0.28). The value of the Pearson correlation coefficient was -0.18. In other words, this correlation was inverse and weak, meaning that with increasing work experience, the number of MN in the micronucleated cells will decrease; however, this reduction was not significant (Table 2 and Figure 3).

**The Relationship Between Exposure Times During the Day (in Hours) and Mean Frequency of MN in the Buccal Mucosa**

In the present study, the maximum exposure time was 5 hours per day, and the minimum was 2 minutes.

**Fig. 4:**
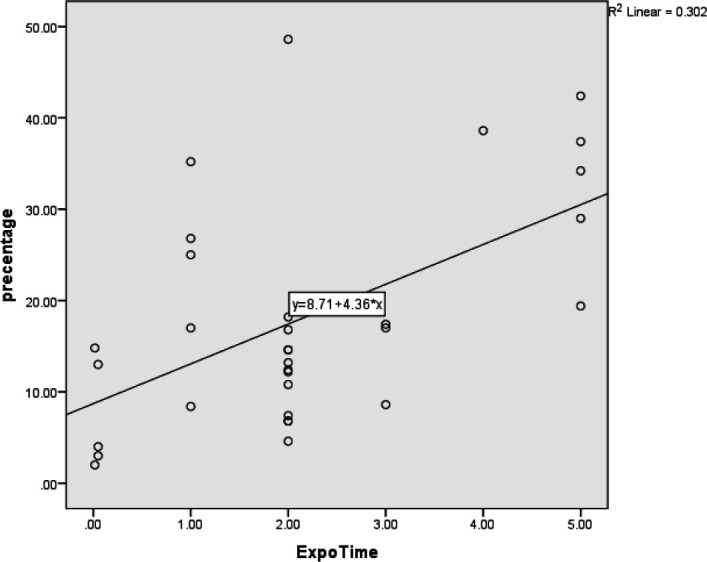
The relationship between exposure times during the day with the mean frequency of micronuclei in the buccal mucosa

The Pearson correlation coefficient showed that the duration of exposure during the day was significantly associated with the mean frequency (mean percentage) of MN in the buccal mucosa (*P*=0.03). The value of the Pearson correlation coefficient was 0.55. In other words, this correlation was direct and strong, meaning that with increasing duration of exposure in a day, the percentage of MN in the buccal mucosa will increase significantly (Table 3 and Figure 4).

**The Relationship Between Exposure Times During the Day (in Hours) and Mean Numbers of MN in the Micronucleated Cells**

**Fig. 5:**
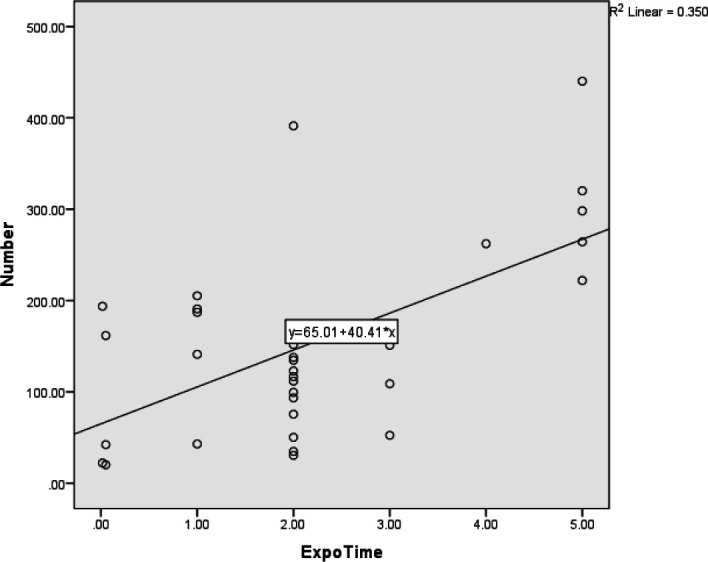
The relationship between exposure times during a day (hourly) with the average number of micronuclei in the cells containing micronucleus

The Pearson correlation coefficient showed that the duration of exposure to formaldehyde during the day (in hours) was significantly associated with the number of MN in micronucleated cells (*P*=0.03). The value of the Pearson correlation coefficient was 0.59, showing a direct and strong correlation. This means that with increasing duration of exposure to formaldehyde, the number of MN in the micronucleated cells will increase significantly (Table 3 and Figure 5).

**Table 3:** The relationship between exposure times during a day (hourly) with micronuclei in the buccal mucosa

Number of MN in the Cells Containing MN		Percentage of the Cells Containing MN		Exposure Time During a Day
P-value	Mean±SD	P-value	Mean±SD	
**0.03**	2.12±0.24	0.03	7.36±5.49	**0-1 hours**
	1.45±0.87		22.48±10.18	**1-2 hours**
	2.04±1.45		14.38±13.21	**2-3 hours**
	1.43±1.98		14.33±4.96	**3-4** **hours**
	1.35±0		38.6±0	**4-5 hours**
	1.90±1.87		32.48±8.78	**≤ ** **5hours**

SD: Standard Deviation

**The Relationship Between Gender and Mean Frequency of MN in the Buccal Mucosa**

In this study, a total of 42 women and 22 men participated, and the 2 groups were matched in terms of gender.

According to the results of the independent *t* test, the difference in the mean frequency (percentage) of MN in the buccal cells in women and men was not significant in all samples (*P*=0.25; Table 4). 

**The Relationship Between Gender and Mean Number of MN in the Micronucleated Cells**

According to the results of the independent* t* test, the difference in the mean number of MN in micronucleated buccal cells in women and men was not significant in all samples (*P*=0.102; Table 4).

**Table 4:** The relationship between gender and micronuclei in the buccal mucosa

MN in the Cells Containing MN			Percentage of Cells Containing MN				Gender
P-value	t	Mean±SD	P-value	t	Mean±SD	Number	
**0.102**	1.65	1.80**±**1.49	0.25	1.16	12.24**±**8.23	22	**Male**
		1.91**±**1.65			15.43**±**11.36	42	**Female**

SD: Standard Deviation

**The Relationship Between Age and Mean Frequency of MN in the Buccal Mucosa**

**Fig. 6:**
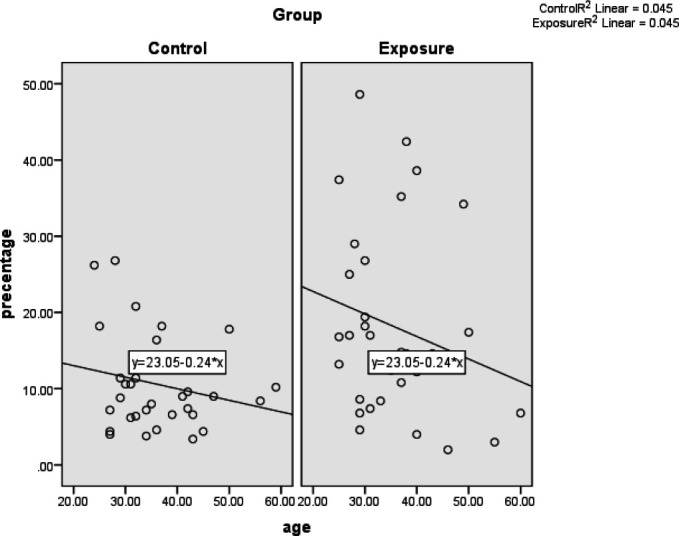
The relationship between ages of individuals with mean frequency of micronuclei in the buccal mucosa

The Pearson correlation coefficient showed no significant relationship between age and percentage of MN in both the case and control groups (*P*=0.32 and *P*=0.39). The Pearson correlation coefficient was -0.212 in the case group and -0.213 in the control group. In other words, this correlation was inverse and weak in both groups. This means that with increasing age, the percentage of MN will decrease in both the case and the control groups, which is not significant (Table 5 and Figure 6).

**Table 5:** The relationship between age of individuals and micronuclei in the buccal mucosa

MN in the Cells Containing MN		Percentage of the Cells Containing MN		Age
Mean±SD				
Control	Case	Control	Case	
**0**	0	0	0	**0-20**
**2.09** **±1.81**	1.73±1.64	8.54±13.37	14.25±20.7	**20-30**
**2.28** **±2.20**	2.09±1.83	5.36±10.06	10.42±18.49	**30-40**
**2.59** **±2.43**	1.44±1.35	3.31±7.92	14.4±21.12	**40-50**
**2.86** **±1.64**	1.25±1.23	6.55±8.66	10.1±10.2	**50-60**
**0**	2.11±0	0±72	0±6.8	**≤** **60**
**0.34**	0.21	0.39	0.32	**p-value**

SD: Standard Deviation

**The Relationship Between Age and Mean Number of MN in the Micronucleated Cells**

**Fig. 7:**
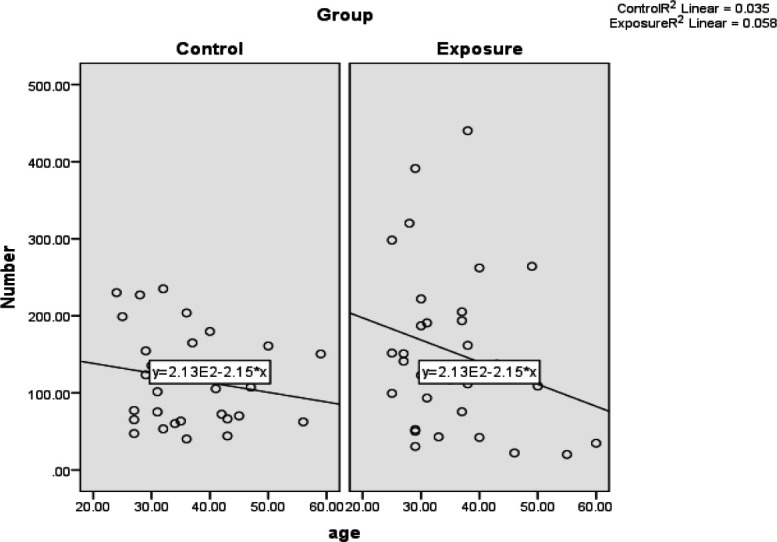
The relationship between age of the individuals and mean number of the micronuclei in cells containing micronuclei

The Pearson correlation coefficient showed no significant relationship between age and number of MN in the micronucleated cells in both case and control groups (*P*=0.21 and *P*=0.34). The Pearson correlation coefficient was -0.19 in the case group and -0.24 in the control group. In other words, this correlation was inverse and weak in both groups. This means that with increasing age, the number of MN in micronucleated cells will decrease in both the case and control groups, which is not significant (Table 5 and Figure 7).

## Discussion

The present study was conducted on 64 individuals who were equally divided into case and control groups (n=32 per group). A total of 500 cells per sample were counted, and the mean frequency of MN was determined. In addition, the mean numbers of MN in micronucleated cells were also counted.

The results showed that the mean frequency of MN was significantly higher in the case group than in the control group (*P*=0.003). This result is consistent with the results found in several previous studies (12, 22, 23, 25, 26, 27). However, it is worth noting that the observed numbers were significantly higher than those reported in similar studies. This discrepancy could be due to the presence of other genotoxic factors, such as air pollution (presence of NO_2_) and exposure to ionizing radiation, both of which have the potential to increase the frequency of MN. Although these genotoxic factors can affect our results, it was not possible to evaluate these factors in the current research. 

Bouraoui *et al.* (2013) used the combination of MN assay and fluorescence in situ hybridization (FISH) to determine the genotoxicity of formaldehyde in peripheral lymphocytes of 31 anatomy and pathology laboratory workers and 31 unexposed individuals. The results showed a significant increase in MN frequency in the case group (25.35±6.28) than in the control group (7.08±4.62; *P*<0.05). This result is consistent with our study, but the number of MN was higher in Bouraoui *et al.*’s study. This difference may be due to the fact that in Bouraoui *et al.*'s study, the FISH technique was also used to evaluate chromosomal damage in addition to MN, which increased the sensitivity and specificity of the results (28).

Fenech *et al.* conducted a systematic review of the harmful effects of human exposure to formaldehyde in 2016. They reviewed 17 articles in which the lymphocyte cytokinesis-block MN (L-CBMN) assay was used. Their results showed that majority of the studies (62%) demonstrated a significant increase in lymphocyte MN frequency in the exposed group compared to the control group (29).

In 2016, Peteffi *et al.* performed a study on 50 employees who were exposed to formaldehyde in 6 beauty salons in Brazil. These salons (except for 2 salons) used hair straighteners that contained formaldehyde. Their results revealed no significant difference in MN, bi-nucleated cells, or nuclear buds between these salons. In contrast, karyorrhexis showed a significant difference (30). The reason for this difference in Peteffi *et al.*’s study may be due to the number of procedures performed each day in each salon and the difference in the amount of exposure to formaldehyde (depending on the type of substance used) in various salons.

In our study, the relationship between years of laboratory work (years of exposure) and average percentage of buccal MN was not significant (*P*=0.17), which is similar to the results of Costa *et al.* in 2008 (25). In contrast, Costa *et al.* in 2013 found a significant correlation between years of exposure and mean percentage of buccal MN, which is consistent with the findings of Lorenzoni *et al.*, Souza and Devi, Sivasankari *et al.*, Neelima and Ravi, Abdel Aziz *et al.*, Souza and Devi, Bouraoui *et al.*, and Fenech *et al.*; these findings are all inconsistent with our results (3,5,12,19,21,22,28,29). The reason for this discrepancy could be due to the fact that most pathologists decrease their working time, and this can impress our findings. Another possible reason for this difference may be an adaptation of various systems of the body over time.

 In addition, Sivasankarinatarajan *et al.* found a highly significant relationship between years of exposure and some nuclear abnormalities, such as karyorrhexis, karyolysis, and bi-nucleated cells, but they did not evaluate MN (31).

In the present study, the relationship between exposure time during the day and mean percentage of MN in the buccal cells was significant (*P*=0.03), which is consistent with the findings of Abdel Aziz *et al.* (19). Furthermore, Sivasankarinatarajan *et al.* revealed a positive correlation between time of exposure and carcinogenesis (31). The results of these studies could indicate that the number of MN increases with extended exposure time during the day (19,31).

In our study, the frequency of MN was higher in women than in men, but the relationship between gender in both case and control groups and mean percentage of the MN was not significant (*P*=0.25). This result is consistent with the findings of Abdel Aziz *et al.* (19) and Costa *et al.* (25).

Furthermore, in the present study, there was no significant relationship between age in both case and control groups and mean percentage of the MN in the buccal mucosa (*P*=0.38), which is consistent with the results of Souza and Devi and Costa *et al.* (2008) (22, 25), but inconsistent with the results of Sivasankari *et al.*, Abdel Aziz *et al.*, Costa *et al.* (2013), and Bouraoui *et al.* (3, 19, 23, 28). The reason for this discrepancy can be due to sampling from different communities; for instance, Sivasankari *et al.*’s study included first-year students and people working in the anatomy laboratory. As it is known, in addition to younger age and less exposure to genotoxic substances during their life, the first-year residents also had less exposure to formaldehyde. 

**In the current study, there was no significant difference between case and control groups in terms of the average number of MN in the micronucleated cells (** ***P*** **=0.11). The relationship between laboratory time of service (individual work experience by year) and total number of MN in the micronucleated cells was not significant (** ***P*** **=0.28). The relationship between time of exposure during the day and mean number of MN in the micronucleated cells showed a significant result (** ***P*** **=0.03). No significant difference was found in the mean numbers of MN in the micronucleated between female and male patients in case, and control groups and all the samples (** ***P*** **=0.102). Moreover, the relationship between age in both case and control groups and all the samples with the total numbers of MN in the micronucleated cells was not significant (** ***P*** **=0.37). According to the literature, previous studies focusing on those individuals working in the pathology laboratories or those exposed to formaldehyde have mainly assessed the frequency of MN in the buccal mucosa (** 3 **,** 5 **,** 19 **-** 23 **,** 25 **), and the numbers of MN in the micronucleated cells have not yet been evaluated. Therefore, it is likely that our study to be the first to investigate this aspect, providing valuable insights for future research in this field. One of the limitations of this study was absence of these data in other studies. Furthermore, due to the COVID-19 pandemic, obtaining samples from subjects was risky and was associated low cooperation.**

## Conclusion

Formaldehyde exposure, particularly extended exposure time during the day, can increase the number of MN, which can prognosticate the incidence of precancerous and cancerous lesions. Therefore, continuous exposure to formalin can be considered as an occupational health hazard, although further studies are needed to confirm this relationship.

## Funding

This research received no specific grant from any funding agencies in public, commercial, or not-for-profit sectors.

## Conflict of Interest

There are no conflicting interests.

## References

[1] Villadiego-Molinares MM, Ramirez-Martinez JA, Rodriguez-Pulido AI (2020). Formaldehyde in occupational environments: literature review and an occupational health surveillance proposal. Rev Fac Med..

[2] Peteffi GP, da Silva LB, Antunes MV, Wilhelm C, Valandro ET, Glaeser J (2016). Evaluation of genotoxicity in workers exposed to low levels of formaldehyde in a furniture manufacturing facility. Toxicol Ind Health..

[3] Sivasankari NP, Sundarapandian S, Sakthivel E (2018). Micronucleus assay in formalin exposed individuals. Indian J Clin Anat Physiol..

[4] Karami Mosafer A, Assari MJ, Bahrami A, Zolhavarie M (2017). Relationship of Ambient Concentrations with Personal Exposure Level of Formaldehyde in the Pathology Departments of Teaching Hospitals Affiliated to Hamadan University of Medical Sciences, Hamadan, Iran. J Occup Hyg Eng..

[5] Lorenzoni DC, Pinheiro LP, Nascimento HS, Menegardo CS, Silva RG, Bautz WG (2017). Could formaldehyde induce mutagenic and cytotoxic effects in buccal epithelial cells during anatomy classes?. Med Oral Patol Oral Cir Bucal..

[6] Lin D, Guo Y, Yi J, Kuang D, Li X, Deng H (2013). Occupational exposure to formaldehyde and genetic damage in the peripheral blood lymphocytes of plywood workers. J Occup Health..

[7] Yahyaei E, Majlesi B, Naimi joubani M, Pourbakhshi Y, Ghiyasi S, Jamshidi Rastani M, Heidari M (2020). Occupational Exposure and Risk Assessment of Formaldehyde in the Pathology Departments of Hospitals. Asian Pac J Cancer Prev..

[8] Bolognesi C, Fenech M (2019). Micronucleus comet assays in human lymphocytes and buccal cells. Methods MolBiol..

[9] Shahsavari F, Ghasemi S, Farhadi S, Mazinani P, Delavari S (2016). Occupational Exposure in Dental Laboratory Technicians May Induce Nuclear Abnormalitiesin Buccal Mucosa Cells: A Preliminary Study. J Res Dentomaxillofac Sci..

[10] Flores-Garcia A, Torres-Bugarin O, Velarde-Félix JS, Rangel-Villalobos H, Zepeda-Carrillo EA, Rodríguez-Trejo A (2014). Micronuclei and other nuclear anomalies in exfoliated buccal mucosa cells of Mexican women with breast cancer. J BUON..

[11] Mørck TA, Loock KV, Poulsen MB, Siersma VD, Nielsen JKS, Hertel O (2016). Micronucleus frequency in Danish schoolchildren and their mothers from the DEMOCOPHES population. Mutagenesis.

[12] Souza AD, Devi R (2014). Cytokinesis blocked micronucleus assay of peripheral lymphocytes revealing the genotoxic effect of formaldehyde exposure. Clin Anat..

[13] Dave G, Parikh N, Patel N, joshi H (2019). Evaluation of Micronucleus Frequencies in Exfoliated Buccal Cells of Potentially Malignant Disorder Subjects Using Feulgen Stain. Indian J Dent Adv..

[14] Sommer S, Buraczewska I, Kruszewski M (2020). Micronucleus Assay: The State of Art, and Future Directions. Int J Mol Sci.

[15] Ceppi M, Biasotti B, Fenech M, Bonassi S (2010). Human population studies with the exfoliated buccal micronucleus assay: statistical and epidemiological issues. Mutat Res..

[16] Shahsavari F, Mikaeli S, Ghorbanpour M (2022). Micronucleus assay in the exfoliated cells of buccal mucosa of gasoline station workers in Tehran. J Can Res Ther..

[17] Hopf N, Bolognesi C, Danuser B, Wild P (2019). Biological monitoring of workers exposed to carcinogens using the buccal micronucleus approach: A systematic review and meta-analysis. Mutat Res Rev Mutat Res..

[18] Aglan M N, Mansour Gh (2020). Hair straightening products and the risk of occupational formaldehyde exposure in hairstylists. Drug Chemic Toxicol..

[19] Abdel Aziz MH, Metwally EA, Zaki EIA A, Azzaz O, Hussein H (2021). Is duration of exposure a determinant factor for genotoxicity and clinical manifestations induced by Formaldehyde?. Ain Shams J Forensic Med Clin Toxicol..

[20] Costa S, Costa C, Madureira J, Valdiglesias V, Teixeira-Gomes A, de Pinho PG (2019). Occupational exposure to formaldehyde and early biomarks of cancer risk, immunotoxicity and susceptibility. Environ Res..

[21] Neelima P, Ravi S (2019). Micronuclei assay in buccal smear of formalin exposed individuals. Indian J Basic Appl Med Res.

[22] Souza AD, Devi R (2014). Micronucleus assay on buccal cells: an indicator of DNA damage due to formaldehyde exposure in anatomy dissection labs. Int J Res Med Sci..

[23] Costa S, Brandao F, Coelho M, Costa C, Coelho P, Silva S (2013). Micronucleus frequencies in lymphocytes and buccal cells in formaldehyde exposed workers. WIT Transactions on Biomedicine Health..

[24] Tolbert PE, Shy CM, Allen JW (1992). Micronuclei and other nuclear anomalies in buccal smears: methods development. Mutat Res..

[25] Costa S, Coelho P, Costa C, Silva S, Mayan O, Santos LS (2008). Genotoxic damage in pathology anatomy laboratory workers exposed to formaldehyde. Toxicology..

[26] Ladeira C, Viegas S, Carolino E, Gomes MC, Brito M (2013). The influence of genetic polymorphisms in XRCC3 and ADH5 genes on the frequency of genotoxicity biomarkers in workers exposed to formaldehyde. Environ Mol Mutagen..

[27] Ladiera C, Veigas S, Carolino E, Prista T, Gomes MC, Brito M (2011). Genotoxic biomarkers in occupational exposure to formaldehyde The case of histopathology laboratories. Mutat Res..

[28] Bouraoui S, Mougou S, Brahem A, Tabka F, Ben Khelifa H, Harrabi I (2013). A combination of micronucleus assay and fluorescence in situ hybridization analysis to evaluate the genotoxicity of formaldehyde. Arch Environ Contam Toxicol..

[29] Fenech M, Nersesyan A, Knasmueller S (2016). A systematic review of the association between occupational exposure to formaldehyde and effects on chromosomal DNA damage measured using the cytokinesis-block micronucleus assay in lymphocytes. Mutat Res Rev Mutat Res..

[30] Peteffi GP, Antunes MV, Carrer C, Valandro ET, Santos S, Glaeser J (2016). Environmental and biological monitoring of occupational formaldehyde exposure resulting from the use of products for hair straightening. Environ Sci Pollut Res Int..

[31] Sivasankari Natarajan P, Subramanian S, Elayaperumal S (2019). Formalin induced nuclear abnormalities in relation with duration of exposure: a study of formalin exposed anatomists, attenders and students. Int J Anat Res..

